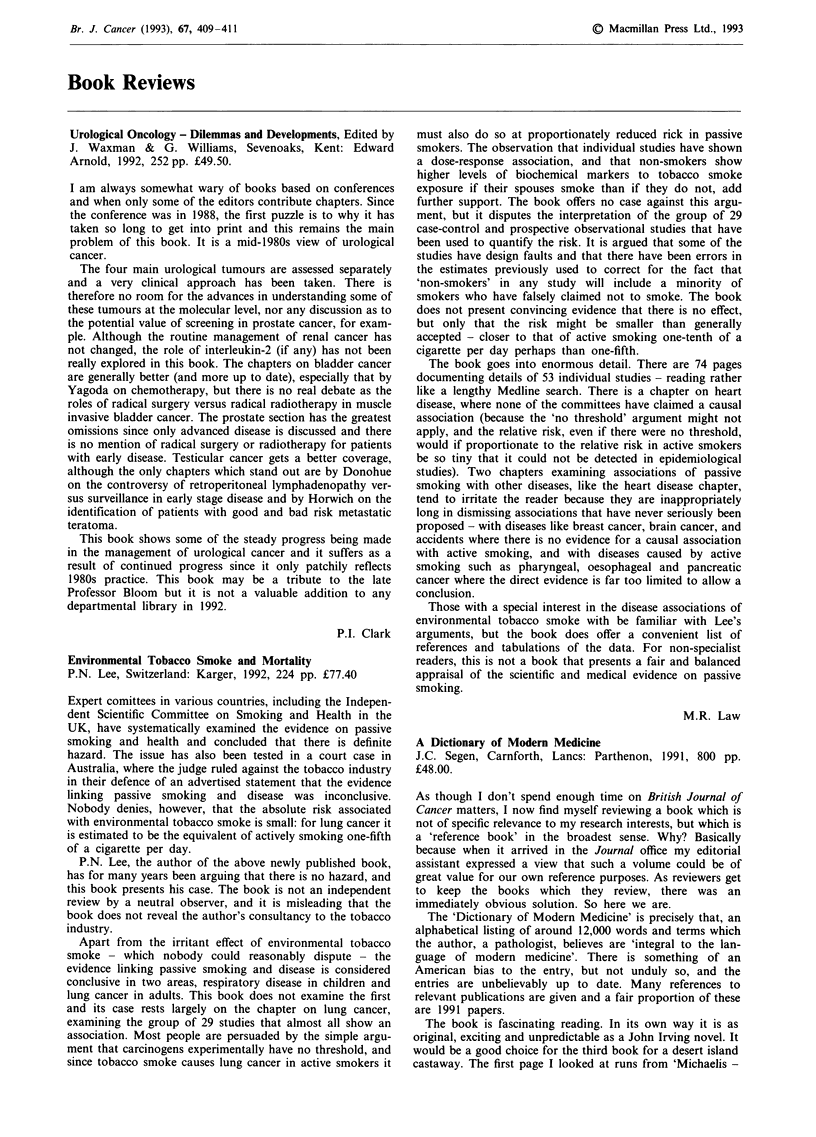# Urological Oncology - Dilemmas and Developments

**Published:** 1993-02

**Authors:** P.I. Clark


### Book Reviews

**Urological Oncology – Dilemmas and Developments**, Edited by J. Waxman & G. Williams, Sevenoaks, Kent: Edward Arnold, 1992, 252 pp. £49.50.

I am always somewhat wary of books based on conferences and when only some of the editors contribute chapters. Since the conference was in 1988, the first puzzle is to why it has taken so long to get into print and this remains the main problem of this book. It is a mid-1980s view of urological cancer.

The four main urological tumours are assessed separately and a very clinical approach has been taken. There is therefore no room for the advances in understanding some of these tumours at the molecular level, nor any discussion as to the potential value of screening in prostate cancer, for example. Although the routine management of renal cancer has not changed, the role of interleukin-2 (if any) has not been really explored in this book. The chapters on bladder cancer are generally better (and more up to date), especially that by Yagoda on chemotherapy, but there is no real debate as the roles of radical surgery versus radical radiotherapy in muscle invasive bladder cancer. The prostate section has the greatest omissions since only advanced disease is discussed and there is no mention of radical surgery or radiotherapy for patients with early disease. Testicular cancer gets a better coverage, although the only chapters which stand out are by Donohue on the controversy of retroperitoneal lymphadenopathy versus surveillance in early stage disease and by Horwich on the identification of patients with good and bad risk metastatic teratoma.

This book shows some of the steady progress being made in the management of urological cancer and it suffers as a result of continued progress since it only patchily reflects 1980s practice. This book may be a tribute to the late Professor Bloom but it is not a valuable addition to any departmental library in 1992.

P.I. Clark